# ER-phagy in the Occurrence and Development of Cancer

**DOI:** 10.3390/biomedicines10030707

**Published:** 2022-03-18

**Authors:** Huimin Zhou, Kexin Wang, Mengyan Wang, Wenxia Zhao, Conghui Zhang, Meilian Cai, Yuhan Qiu, Tianshu Zhang, Rongguang Shao, Wuli Zhao

**Affiliations:** Key Laboratory of Antibiotic Bioengineering, Ministry of Health, Laboratory of Oncology, Institute of Medicinal Biotechnology, Chinese Academy of Medical Sciences and Peking Union Medical College, Beijing 100050, China; zhouhuimin@imb.pumc.edu.cn (H.Z.); wangkexin@imb.pumc.edu.cn (K.W.); wangmengyan@imb.pumc.edu.cn (M.W.); zhaowenxia@imb.pumc.edu.cn (W.Z.); zhangconghui@imb.pumc.edu.cn (C.Z.); caimeilian@imb.pumc.edu.cn (M.C.); qiuyuhan@imb.pumc.edu.cn (Y.Q.); zhangtianshu@imb.pumc.edu.cn (T.Z.); shaor@imb.pumc.edu.cn (R.S.)

**Keywords:** ER-phagy, unfolded protein response, apoptosis, cancer

## Abstract

As an organelle, the endoplasmic reticulum (ER) is closely related to protein synthesis and modification. When physiological or pathological stimuli induce disorders of ER function, misfolded proteins trigger ER-phagy, which is beneficial for restoring cell homeostasis or promoting cell apoptosis. As a double-edged sword, ER-phagy actively participates in various stages of development and progression in tumor cells, regulating tumorigenesis and maintaining tumor cell homeostasis. Through the unfolded protein response (UPR), the B cell lymphoma 2 (BCL-2) protein family, the Caspase signaling pathway, and others, ER-phagy plays an initiating role in tumor occurrence, migration, stemness, and proliferation. At the same time, many vital proteins strongly associated with ER-phagy, such as family with sequence similarity 134 member B (FAM134B), translocation protein SEC62 (SEC62), and C/EBP-homologous protein (CHOP), can produce a marked effect in many complex environments, which ultimately lead to entirely different tumor fates. Our article comprehensively focused on introducing the relationship and interaction between ER-phagy and cancers, as well as their molecular mechanism and regulatory pathways. Via these analyses, we tried to clarify the possibility of ER-phagy as a potential target for cancer therapy and provide ideas for further research.

## 1. Background

Autophagy is a biological process of cell self-eating and is involved in multiple physiological activities, such as protein quality control, cell senescence, immune response, and apoptosis. It plays an indispensable role in various diseases, including tumors, diabetes, Parkinson’s disease, and viral infections [1,2]. According to the different degradation pathways involved, autophagy can be divided into three types: microautophagy, chaperone-mediated autophagy, and macroautophagy. Microautophagy is a non-selective process that can be induced by nitrogen starvation, namely the substrate can be directly engulfed by the lysosome or lysosomal membrane invagination and can then be degraded by proteases. Chaperone-mediated autophagy is a selective process, which arises under oxidative stress; specifically, the targeted protein with a unique molecular motif translocates to the lysosome for degradation with the help of lysosome-associated membrane protein type 2A (LAMP2A), the heat shock cognate protein of 70 kDa (HSC70), and others, instead of drawing support from the membrane structure [3,4,5]. Macroautophagy is a kind of principal degradation mechanism, contributing to the survival of cells under different stressful conditions. In more detail, the double-layer membrane of the endoplasmic reticulum, mitochondria, endosome, etc., gradually forms an autophagosome, bringing with it the substances that need to be eliminated, and then fuses with the lysosomes to compose an autolysosome, in which acid hydrolases disintegrate both the contents and the inner membrane [6,7]. 

The endoplasmic reticulum (ER) is one of the largest organelles in eukaryotic cells. It participates in many vital activities such as protein synthesis, modification, and transportation, as well as lipid synthesis and Ca2+ storage. Under the harmful conditions of nutrient deficiency, hypoxia, pH changes, and the stress of drugs such as tunicamycin, stimulants will trigger ER-phagy to minimize stress and maintain the continual operation of the ER. In 1973, Bolender first found that ER membranes are removed by double-membrane autophagic vacuoles after stopping phenobarbital treatment in hepatocytes [8]. Later, the term “ER-phagy” was coined for the first time [9]. Currently, ER-phagy can be classified into two main types: One is macroautophagy, namely double-membrane autophagosomes containing cytoplasm fused with lysosomes by means of ER-resident or ER-associated receptors. The other is micro-ER-phagy, where cytoplasmic material is directly engulfed by endosomes or lysosomes without receptors or the formation of autophagosomes [10,11,12].

An increasing amount of evidence shows that ER-phagy is associated with the occurrence and development of cancer. For instance, ER-phagy can improve ER function to cope with hypoxia and other destructive stimuli, thus helping tumor cells survive. At the same time, ER-phagy can also participate in tumor cell invasion, metastasis, and drug resistance. It has been found that the inhibition of ER-phagy can restore the sensitivity of tumor cells to chemotherapy drugs and hinder their migration capacity [13,14,15,16]. In contrast, ER-phagy also has a suppressive effect on tumor cells, such as inducing apoptosis, inhibiting stemness, and hindering migration. For example, when the ER-phagy-related gene BECN1 is mutated or knocked out, the expression level of the tumor suppressor gene p53 is significantly reduced, and the probability of tumor occurrence increases [17,18]. In view of the complex effect of ER-phagy on cancer, a clear understanding of the molecular mechanism of ER-phagy and its role in the development of tumors will assist us in discovering new drug targets and treatment options.

In this review, we introduce the regulatory pathways of ER-phagy and its different influences on tumorigenesis and evolution, with the aim of providing new ideas for further research.

## 2. ER-phagy-Related Receptors and Their Protumor or Antitumor Role

ER-phagy receptors are proteins distributed in different regions of the ER and usually have interaction domains that bind to autophagy related protein 8 (Atg8), whose homologues in mammalian cells are microtubule-associated protein light chain 3 (MAP1LC3 or LC3) and gamma-aminobutyric acid receptor-associated protein (GABARAP). Via the recognition and binding of ER-phagy receptors by the abovementioned target molecules, the specific protein is encapsulated in autophagosomes and finally degraded by lysosomes [19]. 

At present, six kinds of ER-phagy receptors anchored in the membrane have been found in mammalian cells: the family with sequence similarity 134 member B (FAM134B), translocation protein SEC62 (SEC62), reticulon 3 (RTN3), cell-cycle progression gene 1 (CCPG1), atlastin GTPase 3 (ATL3), and testis-expressed protein 264 (TEX264). Moreover, there are three soluble ER-phagy receptors in mammals: calcium-binding and coiled-coil domain-containing protein 1 (CALCOCO1), CDK5 regulatory subunit-associated protein 3 (C53), and sequestosome 1 (p62/SQSTM1). It is speculated that FAM134B, CCPG1, and TEX264 are receptors for common ER-phagy in tumor development, while SEC62 may be more significant in the recovery process after anticancer compound treatment, and RTN3L may have a hand in the degradation of the tubular ER. As for ATL3, it is involved in ER-phagy induced by nutrition in tumors [20,21]. 

**Figure 1:**
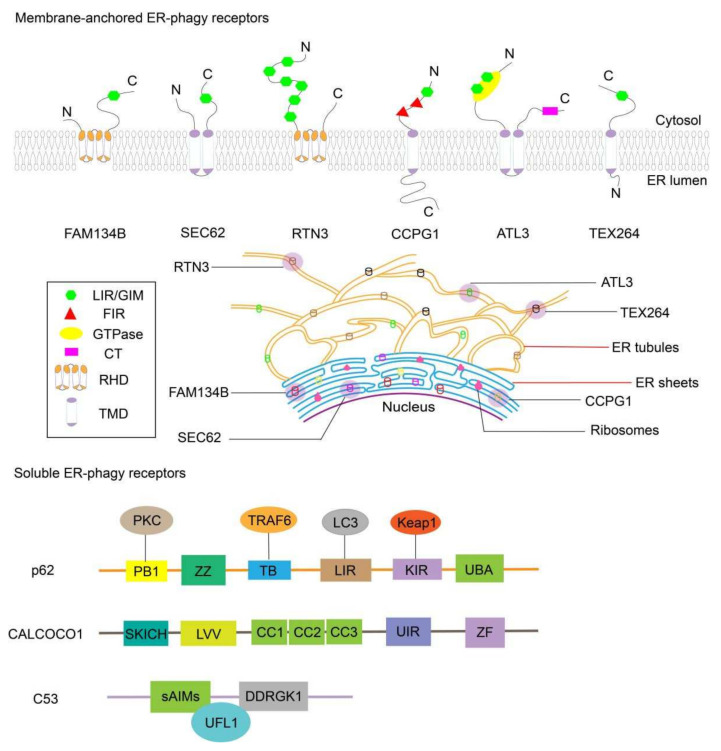
The essential receptors of ER-phagy. Generally, FAM134B, SEC62, and CCPG1 are located in the ER sheets, while RTN3, ATL3, and TEX264 exist in the tubular ER; meanwhile, p62, CALCOCO1, and C53 are three soluble receptors that are distributed in different regions. Although they have various functional motifs and locations, they all play indispensable roles in ER-phagy and have unique interactions with the development of cancer. For instance, FAM134B and RTN3 are activated upon amino acid deprivation, while SEC62 and CCPG1 play more roles in ER-phagy induced by drugs. In its relationship with tumors, FAM134B can inhibit tumor cell migration, while SEC62 can promote the invasion and metastasis ability of cancer cells. The other functions of these receptors in ER-phagy and cancer require further research.

Herein, we tried to summarize some basic information regarding these receptors, and a schematic is provided in Figure 1, although more research is needed on their physiological functions and regulatory mechanisms in ER-phagy.

### 2.1. FAM134B

FAM134B is the first ER-phagy receptor to have been studied. It contains a reticulon-homology domain (RHD) at the N-terminus, which helps ER fragments be divided and then formed into autophagic vesicles. At the same time, FAM134B has an LC3-interacting region (LIR motif) at the cytosolic C-terminus, which can specifically bind to the LC3 or GABARAP of autophagosomes [22]. Synergistically with autophagy related protein 5 (Atg5), Beclin-1, and focal adhesion kinase family interacting protein of 200 kDa (FIP200), FAM134B participates in the process of ER-phagy. A change in the expression of FAM134B may lead to the dilation and dysfunction of the ER, causing cancer and other disorders [22,23,24]. 

In the study of Shohei Kohno, they found that starvation promotes FAM134B-2 gene expression by the induction of the transcription factor CCAAT/enhancer-binding protein beta (C/EBPβ). FAM134B-2 is an N-terminally truncated isoform of FAM134B and can also interact with LC3 and modulate lysosomal degradation. It is speculated that FAM134B-2 may be an important contributor of selective ER-phagy mediated by nutritional deficiency in tumor formation [25]. 

Compared with nontumor tissues, FAM134B is upregulated in hepatocellular carcinoma (HCC), as well as being associated with tumor proliferation and metastasis via the AKT and GSK3-β signaling pathways. Meanwhile, the downregulation of FAM134B leads to an increase in the growth rate and reduces apoptosis in colorectal carcinoma, due to the over expression of end-binding protein 1 (EB1) and the translocation of β-catenin to the nucleus [26,27,28]. Further details on the relationship between FAM134B and cancers are provided in Section 3.2.2.

### 2.2. SEC62

SEC61, SEC62, and SEC63 are ER transmembrane proteins, which collectively play a central role in the translocation of newly synthesized precursor polypeptides into the ER. With different topological domains, SEC61, SEC62, and SEC63 have various functions, and only SEC62 has a close relationship with ER-phagy [29]. SEC62, with an LIR at the cytosolic C-terminus, can connect to LC3 on the autophagosome membrane to promote the migration of autophagosomes to lysosomes. By removing specific ER fragments, SEC62 can maintain the volume and size of the ER [23,30]. It has been reported that the SEC62-driven return of the ER size needs the endosomal sorting complex required for transport-III (ESCRT-III) component CHMP4B and the accessory AAA+ ATPase VPS4A, which are involved in membrane repair, remodeling, and fission events [31,32].

In non-small cell lung, prostate, and thyroid cancers, the increased expression of SEC62 can enhance the cellular tolerance of ER stress and can promote the invasion and metastasis ability of cancer cells [33]. In human colorectal cancer, upregulated SEC62 enhances cancer stemness and progression through triggering Wnt/β-catenin signaling and is associated with the poor prognosis in patients [34]. Under hypoxia, SEC62 can also promote angiogenesis in hepatocellular carcinoma [35].

### 2.3. RTN3

There are four kinds of RTN family proteins: RTN1, RTN2, RTN3, and RTN4. Under each type, there are some isoforms with different lengths. Their common feature is that they all contain RHD at the C-terminal domain, while the N-terminal domain exists in different sites [23,36]. 

During ER-phagy, only RTN3L, the longest isoform of RTN3, participates in this process. The N-terminal cytosolic domain of RTN3L contains six LIRs, which are activated by starvation to form an RTN3L dimer complex. This dimer complex induces part of the tubular ER to be fragmented, which is then delivered to lysosomes to be degraded [36,37]. 

It should be noted that FAM134B preferentially interacts with RTN2L rather than RTN3L, which may be bound up with the fact that FAM134B is mainly located at the edge of ER sheets and RTN3 is located in ER tubules [36,37,38]. 

Recent studies have found that the RTN-1C protein in this family is closely related to LC3 and Atg16L1 during ER-phagy and can be co-localized on the autophagosome membrane. It has been speculated that RTN-1C is involved in the formation of autophagosomes and may have similar functions to other ER-phagy receptors [39]. 

Shushu Song found that compared to normal hepatocytes, RTN3 is poorly expressed in HCC and acts as a tumor suppressor by driving growth arrest in vitro and in vivo by facilitating p53 Ser392 phosphorylation through Checkpoint kinase 2 (Chk2); however, the role of RTN3 in cancers still requires adequate experimentations [40].

### 2.4. CCPG1

Differently from the above three receptors, CCPG1 is an unconventional ER-phagy receptor, consisting of three parts: the N-terminal cytosolic domain, the longer C-terminal domain in the ER lumen, and a transmembrane domain anchored in the ER membrane. The N-terminal cytoplasmic domain contains an LIR and two FIP200-interacting regions (FIR for short). It is speculated that the LIR and FIR can recruit autophagosome membranes, and the C-terminal domain can recognize and interact with misfolded proteins [37,41]. 

In cells lacking CCPG1, the RTN3-mediated tubular ER fragmentation process is impaired during ER-phagy induced by nutrient deprivation, while the degradation process of ER sheets mediated by FAM134B is not affected, which may indicate that CCPG1 has a synergistic effect with RTN3 rather than FAM134B [23,41].

At present, CCPG1 has been found to play an important role in inhibiting the accumulation of proteins in the ER and preventing the overreaction of UPR and may have a close relationship with pancreatic cancers [38,41]. CCPG1 also acts as a tumor suppressor in retinoblastoma, probably related to cell apoptosis and proliferation [42]. Further research on CCPG1 in cancer is required.

### 2.5. ATL3

Atlastins (ATLs) are GTPases that reside in the ER, which mainly include ATL1, ATL2, and ATL3. Structurally, ATLs can be divided into three parts: an N-terminal GTPase followed by two closely spaced transmembrane segments and a C-terminal tail (CT). Though the expression level of ATLs varies significantly in different cellular environments, they have complementary functions due to their highly similar sequences during ER-phagy in tumor occurrence [43,44]. 

Among different regions, N-terminal GTPase can undergo transdimerization mediated by GTP binding and conformational changes induced by GTP hydrolysis, which is of great significance in the fusion reaction carried out by ATLs [45]. Moreover, ATL3 contains two GABARAP-interacting motifs (GIMs) on the N-terminus of the long chain, which can specifically bind to GABARAP, but cannot bind to LC3 subfamily proteins [46]. During ER-phagy, ATL3 can induce the degradation of proteins in the tubular ER to maintain ER stability. 

Further studies have shown that ATL3 can work synergistically with RTN3L. When ATL3 is deficient, overexpressed RTN3L can make up for the disorder of ER-phagy, and vice versa [44,46]. The relationship between ATL3 and cancer requires further research.

### 2.6. TEX264

TEX264 is one of the most highly expressed proteins in colorectal cancer. It contains an N-terminal luminal region, a C-terminal cytosolic region harboring an LIR, and a cytosolic gyrase inhibitor (GyrI)-like domain connecting the above two regions. The same as RTN3 and ATL3, TEX264 is mainly distributed in the tubular ER [47,48]. In the case of malnutrition, TEX264 connects to Atg8 family proteins through the LIR, preferentially with LC3A, LC3B, and GABARAPL1 in mammalian cells [47]. Haruka Chino found that TEX264 can participate in the formation of autophagosomes and then is trafficked to the lysosome for degradation. 

Studies in HeLa cells have found that after the knocking out of TEX264, FAM134B, and CCPG1, respectively, the deletion of TEX264 is the most effective in inhibiting ER-phagy, which suggests that TEX264 is a major ER-phagy receptor [49]. The relationship between TEX264 and tumors remains to be investigated.

### 2.7. CALCOCO1 

CALCOCO1 is a kind of soluble protein consisting of an N-terminal SKIP carboxyl homology (SKICH) domain, middle coil–coil (CC) regions, and carboxy terminal domains containing zinc finger domains, which are targeted to the ER by interacting with VAPA/B, two ER-membrane-localized proteins via a FFAT-like motif, to mediate ER-phagy under starvation-induced stress. There is an atypical LIR (CLIR) motif (LVV) located at the linker region between the SKICH domain and the CC domain, which can bind to the GABARAP subfamily. Moreover, the C-terminal of CALCOCO1 has a UDS-interacting region (UIR), which can interact with Atg8 family members [50,51,52]. 

Studies have shown that the mRNA and protein levels of CALCOCO1 are depressed in colorectal cancer; it is likely that CALCOCO1 works as a tumor inhibitor, related to cancer cell metastasis [53]. At the same time, CALCOCO1 mutation is a risk with regard to the occurrence of breast cancer [52].

### 2.8. C53 

C53 is a sort of soluble protein that can directly interact with Atg8 via shuffled Atg8-family interacting motifs (sAIMs) located in its intrinsically disordered region (IDR). Notably, C53 is not activated by nutrient starvation or UPR sensors, only by ER stress induction such as ribosome stalling, when C53 is recruited to the ER by forming a ternary receptor complex with its ER membrane adaptor DDRGK domain-containing protein 1(DDRGK1) and the ubiquitin-fold modifier 1-specific ligase 1(UFL1), namely the E3 ligase that mediates ufmylation [54,55]. It has been reported that C53 is overexpressed in HCC, which is involved in tumorigenic activity and metastatic potential [56].

### 2.9. SQSTM1/p62

Different from the abovementioned ER receptors, p62 is a multifunctional protein distributed throughout cells [57]. Structurally, p62 contains six functional motifs: an N-terminal Phox-BEM1 domain (PB1) for protein kinase C (PKC) binding, a central ZZ-type zinc finger domain, a TRAF6-binding domain (TB), an LIR that interacts with LC3, a Keap1-binding region (KIR), and a C-terminal ubiquitin-associated (UBA) domain [58]. 

After being activated by specific proteins, p62 targets and binds to the transmembrane receptor tripartite motif containing 13 (TRIM13) on the ER to form oligomers. TRIM13 can interact with VPS34 and Beclin-1 to induce the curvature and fragmentation of the ER membrane, which, in turn, guides the formation of autophagosomes [59]. p62 can also non-covalently bind to the protein modified by ubiquitin through the UBA domain and can then deliver the cargo to the autophagosome via the LIR domain or to proteasome through the PB1 domain for degradation [57]. 

p62 goes hand in hand with tumorigenesis. The continuous expression of p62 caused by ER-phagy defects promotes tumor growth. Meanwhile, the tumor expansion caused by ER-phagy inhibition disappears upon the knockout of p62 [60].

## 3. The Procancer or Anticancer Roles and Molecular Pathways of ER-phagy 

**Figure 2:**
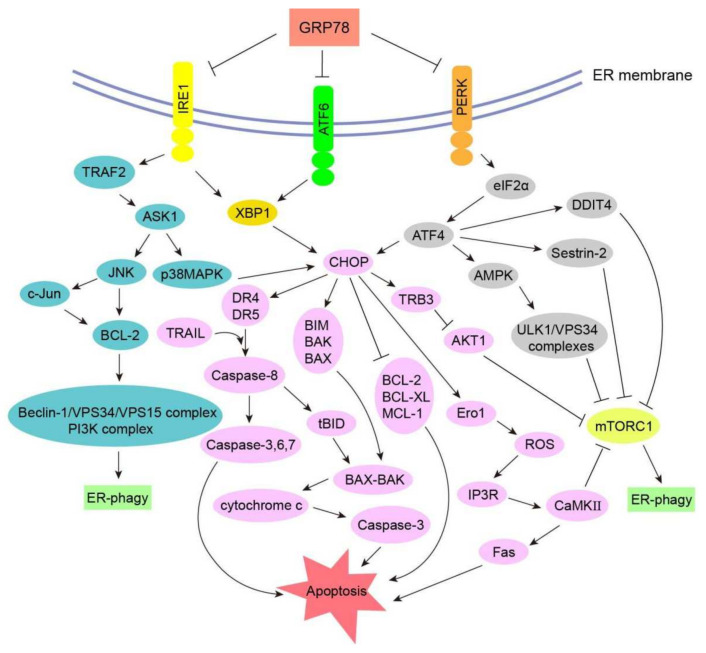
Molecular mechanism of the unfolded protein response and ER-phagy. Under normal physiological conditions, GRP78 can inhibit the activity of IRE1, ATF6, and PERK. Under ER stress, the misfolded proteins can lead to their dissociation from GRP78 and trigger downstream signal propagation. IRE1 interacts with TRAF2 and generates XBP1s, then prompts ER-phagy through the JNK and p38MAPK pathways. ATF6 is transported to the Golgi apparatus and is cleaved to form XBP1s, thus regulating CHOP and its downstream targets, such as the BCL-2 protein family and Caspase signal transduction, resulting in the aggravation of ER-phagy and cell apoptosis. PERK can phosphorylate eIF2α to inhibit protein synthesis, while ATF4 containing short open reading frames is increased and facilitates CHOP, AMPK, Sestrin-2, and DDIT4 to regulate ER-phagy and apoptosis.

ER stress and ER-phagy play important roles in all periods of cancer development. During initiation and the proceeding stage, due to the rapid proliferation of tumor cells, some adverse conditions such as insufficient ATP, low PH, and a lack of nutrition lead to the ER and other organelles becoming seriously damaged. When UPR is insufficient to cope with unfavorable cellular stress, ER-phagy is triggered through a variety of signal transduction pathways. If the stress intensity is beyond the regulating threshold of ER-phagy or ER-phagy is impaired, the stimulus activates apoptosis proteins to accelerate tumor cell death [61]. During this process, different proteins and signal transduction pathways may lead to completely different cell fates. For example, the 78 kDa glucose-regulated protein/binding immunoglobulin protein (GRP78/BiP) can promote tumor cell survival and metastasis through the phosphatidylinositol 3-kinase (PI3K)/AKT signaling pathway; spliced X-box binding protein 1 (XBP1s) (a transcription factor) can facilitate angiogenesis; activating transcription factor 6 (ATF6) can induce drug resistance. At the same time, the protein kinase R-like ER kinase (PERK)/CHOP signaling pathway can motivate cell apoptosis; phosphorylated eukaryotic initiation factor 2α (p-eIF2α) can weaken tumor invasion; the deletion of Beclin-1 can result in the initiation of a tumor [62,63]. In the following section, we summarize the dual procancer and prodeath role of ER-phagy in cancer cells, and a schematic diagram of the unfolded protein response and ER-phagy is shown in Figure 2.

### 3.1. The Procancer Role of ER-phagy

#### 3.1.1. ER-phagy Is Advantageous to the Invasion and Metastasis of Tumor Cells

Cancer cells can spread to normal tissues and form new tumors, and ER-phagy is involved in regulating this process in many ways. For instance, the activation of PERK can promote the metastasis of tumor cells by increasing the expression of vascular endothelial growth factor A (VEGFA), mediating lysosomal-associated membrane protein 3 (LAMP3), and inducing the activity of disintegrins and metalloproteases [64,65,66]. In the study of Maximilian Linxweiler, they found that SEC62 silencing inhibits the capability of migration in lung and thyroid cancer cells without affecting cell proliferation, while nonmigrating human embryonic kidney cells overexpressing SEC62 show stronger migration potentiality, indicating that SEC62 is essential for tumor cell metastasis and invasion [67]. Patricia Dauer found that compared to control cells, the knockout of GRP78 in pancreatic cancer cells not only inhibits cell proliferation, but also affects cell migration and invasion performance, which illustrates that GRP78 also has a promoting effect on tumor cells [68].

#### 3.1.2. ER-phagy Mediates Drug Resistance in Tumor Cells

Cancer patients that constantly receive chemotherapy will elicit drug resistance. At present, it is known that there are many factors resulting in this phenomenon, including increased drug efflux, drug inactivation, and a change in drug targets. From the perspective of ER-phagy, it can participate in regulating this process by virtue of GRP78, ATF6, activating transcription factor 4 (ATF4), and inositol-requiring enzyme 1 (IRE1) [69]. 

Studies have shown that in a model of athymic nude mice, paclitaxel treatment inhibits the growth of breast tumors. The combination of an IRE1 inhibitor and paclitaxel could enhance antibreast cancer efficacy. Moreover, when paclitaxel is withdrawn, the IRE1 inhibitor alone can still restrain tumor growth, thus proving that the IRE1 signaling pathway might mediate drug resistance in tumor cells [70].

#### 3.1.3. ER-phagy Facilitates Cancer Cell Survival by Promoting Angiogenesis

In order to adapt to adverse conditions such as insufficient ATP and hypoxia, the expression of vascular endothelial growth factor (VEGF) in tumor cells is enhanced through ER stress responders ATF4 and XBP1s, which then promote the formation of new blood vessels to provide oxygen and other essential nutrients. At the same time, VEGF can also affect ER-phagy through the mammalian target of rapamycin complex 1 (mTORC1) [71]. 

Studies have found that activating the PERK/ATF4 pathway can effectively promote tumor angiogenesis. By silencing ATF4 or PERK to block this signal transduction, the production of pro-angiogenic factors is markedly reduced [72,73].

#### 3.1.4. ER-phagy Protects Tumor Cells by Immunosuppression

When cancer cells are in an extreme environment such as one with excessive reactive oxygen species (ROS), they can recruit immune cells, including dendritic cells and Tc lymphocytes, and disrupt their functions to block their recognition and clearance effects, which can be regulated by ER-phagy [74,75]. For example, tumor cells can inhibit the antigen presentation of immune cells and block the activation of T lymphocytes by activating the IRE1/XBP1 pathway in order to protect themselves from elimination [76]. C/EBP-homologous protein (CHOP), which is overexpressed in tumor cells, can regulate pro-inflammatory cytokines, such as IL-23, IL-1β, and IL-6, and modulate the activity and survival of tumor cells by inhibiting the immune response of T cells [75]. Moreover, phosphorylated eIF2α can reduce the synthesis of major histocompatibility complex (MHC) molecules and can also impair antigen presentation [77].

### 3.2. The Role of ER-phagy in Inhibiting Tumorigenesis

#### 3.2.1. Excessive ER-phagy Contributes to Cancer Cell Death

If the stimulus intensity is too strong or the duration is too long, the adaptive response of the ER is insufficient to restore the normal physiological state of tumor cells, and ER-phagy will activate cell apoptosis through CHOP, Caspase, JUN N-terminal kinase (JNK), and other pathways [78]. Generally, ER-phagy often precedes the occurrence of apoptosis, and there is an antagonistic effect between them.

ER-phagy induced by low-level stimulation can inhibit the activation of apoptosis-related proteins; meanwhile, under high-intensity stimulation, ER-phagy is activated instantaneously. Along with apoptosis, ER-phagy is inhibited and the protein is lysed. Moreover, apoptosis caused by over-activated ER-phagy is an irreversible process. Once started, even if the stimulus disappears, cells cannot return to their original state, which may be related to the destruction of indispensable components of tumor cells [79,80,81].

#### 3.2.2. ER-phagy Inhibits Tumor Cell Migration

At present, it is well known that the ER-phagy receptor FAM134B is related to tumor proliferation, recurrence, and pathological staging [82,83]. Studies have found that after knocking out FAM134B, the migration rate and invasive capacity of colon cancer cells greatly increase, which indicates that FAM134B could inhibit tumor cell migration in tissues and may even prevent cancer cells from forming new metastases [84].

#### 3.2.3. ER-phagy Adversely Affects the Stemness of Tumor Cells

In malignant tumor tissues, some cancer cells cannot be easily killed by drugs and actively participate in tumor growth, metastasis, drug resistance, and recurrence. Such cells are called cancer stem cells (CSCs). CSCs can self-renew and differentiate into various types of cells, which has a profound influence on antitumor therapy [85,86].

Studies have found that by activating the PERK/eIF2α signaling pathway in colorectal cancer cells, the expression of stem cell markers, such as leucine-rich repeat-containing G protein-coupled receptor 5 (LGR5) and olfactomedin 4 (OLFM4), is decreased and translation inhibition is induced by phosphorylated eIF2α, which can lead to cell cycle arrest or apoptosis. In other words, the stemness and proliferation activity of cancer cells is adversely affected by the cross-interaction of the UPR and ER-phagy [87].

### 3.3. Signal Transduction Pathways of ER-phagy in Cancers

#### 3.3.1. CHOP–BCL-2 Protein Family

As a key factor, CHOP plays an important role in regulating the B cell lymphoma 2 (BCL-2) protein family to promote cell apoptosis during ER-phagy. After the activation of PERK induced by ER stress, the subsequently upregulated ATF4 can enhance the expression of CHOP; at the same time, the activated ATF6 and IRE1 also can raise CHOP by XBP1 [88]. As a transcriptional regulator, CHOP can modulate the BCL-2 protein family, namely downregulating the expression of anti-apoptotic proteins such as BCL-2, B cell lymphoma-extra large (BCL-XL), and Myeloid cell leukemia-1 (MCL-1), as well as upregulating pro-apoptotic proteins such as BIM, BCL-2 antagonist killer (BAK), and BCL-2-associated X protein (BAX) [89]. BAK and BAX can assemble into oligomeric complexes, which permeabilize the outer mitochondrial membrane to release cytochrome c and other apoptosis-inducing factors into the cytoplasm, thus activating the downstream signal transduction pathway, such as the Caspase-3 signaling cascade, which ultimately leads to tumor cell death [90].

#### 3.3.2. Caspase Signaling Pathway

Activated PERK induces the expression of ATF4, which then promotes ATF3 and CHOP. CHOP and ATF3 can upregulate death receptor 4 (DR4) and death receptor 5 (DR5) by combining with their gene promoters. Meanwhile, ER stress can trigger the TNF-related apoptosis-inducing ligand (TRAIL), which binds to DR4 and DR5 to regulate Caspase-8 cascades, resulting in the activation of downstream effectors Caspase-3, Caspase-6, and Caspase-7 to accelerate cell apoptosis [91,92]. The activation of Caspase-8 can also cleave the pro-apoptotic protein BH3-interacting domain death agonist (BID) of the BCL-2 family into truncated BID (tBID), which has strong pro-apoptotic activity and can act on the mitochondrial membrane to regulate the BAX-/BAK-mediated mitochondria apoptotic pathway [93]. In addition, the self-oligomerization of IRE1 can induce the activation or upregulation of a variety of pro-inflammatory proteins in cancer cells, thereby boosting the inflammatory response and death signaling pathways that depend on Caspase-1, Caspase-4, and Caspase-12 [94,95]. Meanwhile, IRE1-mediated degradation of anti-Caspase-2 microRNA results in the activation of the apoptotic promoter Caspase-2, which can then trigger the mitochondrial-dependent apoptosis pathway [96]. 

#### 3.3.3. Ero1 Signaling Pathway

ER oxidoreductin 1 (Ero1) is an ER-resident thiol oxidoreductase that can catalyze the formation and isomerization of protein disulfide bonds to promise the correct folding of proteins in the ER, and this catalyzing process brings about the storage of H_2_O_2_ in the ER [97]. CHOP can increase Ero1, and excess H_2_O_2_ leaks into the cytoplasm, which can not only activate JNK, induce pro-apoptotic proteins BAX and BAK, and inhibit anti-apoptotic proteins BCL-2, BCL-XL, and MCL-1 to mediate cell apoptosis, but can also promote ROS accumulation [98]. When the concentration of ROS reaches a high level, it triggers the IP3R calcium channel and transfers calcium ions from the ER to the cytoplasm. Calcium ions can activate JNK and calcium-/calmodulin-dependent protein kinase (CaMK), thus inducing Fas receptors and relieving the inhibitory effect of mTORC1 on the uncoordinated 51 (UNC-51)-like kinase 1 (ULK1) complex. In other words, the Ero1 signaling pathway plays a profound role in both ER-phagy and apoptosis [99].

#### 3.3.4. GADD34 Signaling Pathway

Growth arrest and DNA damage-inducible protein 34 (GADD34) is also one of the downstream targets of ATF4/CHOP. Under stress, this protein can mediate protein phosphatase 1 (PP1) to dephosphorylate eIF2α, so that the translation of proteins can resume; additionally, unfolded or misfolded proteins accumulate in the ER, leading to ATP consumption, oxidative stress, and tumor cell apoptosis [100,101].

#### 3.3.5. ASK1-p38 MAPK/ASK1-JNK Signaling Pathway

The activation of IRE1 can trigger apoptosis signal-regulating kinase 1 (ASK1) and then phosphorylate downstream p38 mitogen-activated protein kinase (p38MAPK) and JNK. p38MAPK can phosphorylate Ser78 and Ser81 of CHOP, resulting in CHOP having an enhanced transcriptional capacity, which is conducive to pro-apoptotic genes such as DR5 and BIM, thereby accelerating apoptosis [102,103]. Phosphorylated JNK can not only promote the destruction of BCL-2 family proteins with mitochondrial protection functions such as BCL-XL and MCL-1, but can also directly activate BAX and BAK proteins with pro-apoptotic activity, which can result in apoptosis through the mitochondrial pathway [94,95]. At the same time, activated JNK can phosphorylate Ser63 and Ser73 of the downstream target c-Jun. As a transcription regulator, activated c-Jun also can modulate BAX and BCL-2, thus promoting cell apoptosis [104].

## 4. ER-phagy as a Potential Target for Cancer Therapy

ER-phagy has a dual effect on tumor cells, which can not only promote their occurrence and development, but can also induce their apoptosis. Therefore, with ER stress and ER-phagy as targets, cancer treatment strategies are mainly classified into two types: one is to block ER-phagy, which is beneficial for cell survival, so as to facilitate tumor cell apoptosis; the other is to increase the stimulus intensity to overload the tumor cell and trigger the death pathways [105]. Accordingly, ER-phagy inducers and inhibitors are equally promising in cancer treatment. A brief introduction is given below.

### 4.1. Activating ER-phagy to Treat Cancer

The induction of ER-phagy can be used as an effective method to accelerate tumor cell death in cancer therapy. For example, disulfiram (DSF) is an anti-alcoholism drug that shows potent anticancer activity. Studies have shown that it could promote ER-phagy by activating IRE1α, which in turn can lead to cellular apoptosis, indicating that it is an attractive candidate in the area of cancer therapeutics [106]. Metformin is prescription drug that is frequently used for the treatment of type 2 diabetes, because it can activate AMPK and inhibit mTORC1, so it can also be used for cancer treatment. At present, it is being tested in clinical trials with several models [107]. At the same time, combination drugs also show good therapeutic prospects. For instance, combining bortezomib with thapsigargin or celecoxib severely aggravates ER stress and greatly exacerbates tumor cell death [108]. Some natural products have been reported to show anticancer activity by inducing ER-phagy. 2-(3,4 dihydroxyphenyl ethanol) ethanol, derived from olive oil, can activate the PERK/eIF2α/CHOP and IRE1/JNK signaling pathways, thus inducing apoptosis of HT-29 cells via ER stress [109]. 

### 4.2. Inhibition of ER-phagy to Treat Cancer

ER-phagy inhibition is gaining attention as a potentially new therapeutic strategy in cancer, which can be sorted into many kinds of types, including lysosomal inhibitors and ATG4B inhibitors. At present, lysosomal inhibitors such as chloroquine (CQ) and hydroxychloroquine (HCQ) have been used clinically to control tumor growth or induce tumor cell death [110]. In animal experiments, the inhibitors of the ULK complex and VPS34 have shown great treatment effect. The IRE1 inhibitor STF083010 and toyocamycin, which can block XBP1s mRNA splicing, have also been widely used in animal studies for tumor treatment [110,111]. Moreover, some new drugs such as VPS18 and VPS34 kinase inhibitors SAR405, HCQ analog Lys05, and PI3K kinase complex inhibitor Spautin-1 are all under research and development [112]. At the same time, a number of tool compounds are undergoing laboratory research, such as lysosomal inhibitors mefloquine and verteporfin and Beclin-VPS34 complex inhibitors 3-methyladenine, LY294002, and Wortmannin [113]. Notably, many newly active drugs are constantly being discovered or synthesized, such as the GRP78 inhibitor epigallocatechin gallate (EGCG), which blocks the formation of a GRP78/Caspase-7 complex and prevents the anti-apoptotic effects of GRP78 [111]. 

## 5. Conclusions

Mild ER stress is one of the defense mechanisms of tumor cells that can help to maintain cell homeostasis. However, a strong stimulus can induce ER-phagy and even lead to apoptosis. In recent years, research on the molecular mechanism of ER stress and ER-phagy has made great progress, and the rational application of ER-phagy to fight cancer is a promising developmental direction. However, there are still some problems that need to be solved urgently, for example: How can ER-phagy be selectively regulated in tumor cells rather than in healthy cells? What is the balance between cell survival and death mediated by ER-phagy? How does the ER cooperate with other organelles to modulate tumor cell death? From our point of view, though we know much about ER-phagy, in order to treat cancer with a more powerful weapon, there remain many mysteries in this field, making it difficult to judge how to utilize ER-phagy most efficiently to treat cancer. Perhaps to the answers to these mysteries might be found by examining the various cancer types, progression stages, cell microenvironments and states, and individual differences. Currently, we can combine other medical technologies with ER-phagy, such as targeted drug delivery systems, biosensors, and artificial cells, to elucidate the regulatory mechanism of ER-phagy and apoptosis induced by ER stress, which will contribute to the provision of new therapeutic strategies for cancer and other diseases.

## Abbreviations

ER	Endoplasmic reticulum
HSC70	Heat shock cognate protein of 70 kDa
LAMP2A	Lysosome-associated membrane protein type 2A
FKB	Flavokawain B
MAP1LC3	Microtubule-associated protein light chain 3
GABARAP	Gamma-aminobutyric acid receptor-associated protein
FAM134B	Family with sequence similarity 134 member B
SEC62	Translocation protein SEC62
RTN3	Reticulon 3
CCPG1	Cell-cycle progression gene 1
ATL3	Atlastin GTPase 3
TEX264	Testis-expressed protein 264
p62/SQSTM1	Sequestosome 1
RHD	Reticulon-homology domain
LIR	LC3-interacting region
HCC	Hepatocellular carcinoma
Chk2	Checkpoint kinase 2
FIR	FIP200-interacting region
ATL	Atlastin
3HB	Three-helix bundle
CT	C-terminal tail
GIMs	GABARAP-interacting motifs
GyrI	Gyrase inhibitor
CALCOCO1	Calcium-binding and coiled-coil domain-containing protein 1
C53	CDK5 regulatory subunit-associated protein 3
UFL1	Ubiquitin-fold modifier 1-specific ligase 1
DDRGK1	DDRGK domain-containing protein 1
PB1	Phox-BEM1
PKC	Protein kinase C
TB	TRAF6-binding
KIR	Keap1-binding region
TRIM13	Tripartite motif containing 13
IRE1	Inositol-requiring enzyme 1
ATF6	Activating transcription factor 6
PERK	Protein kinase R-like ER kinase
GRP78/BiP	78 kDa glucose-regulated protein/binding immunoglobulin protein
LD	Luminal domain
SBD	Substrate binding domain
RNase	Endoribonuclease
XBP1s	Spliced X-box binding protein 1
TNF	Tumor necrosis factor
TRAF2	TNF receptor-associated factor 2
ASK1	Apoptosis signal-regulating kinase 1
JNK	JUN N-terminal kinase
BCL-2	B cell lymphoma 2
PI3K	Phosphatidylinositol 3-kinase
ERp72	ER protein 72
eIF2α	Eukaryotic initiation factor 2α
ATF4	Activating transcription factor 4
CHOP	C/EBP-homologous protein
AMPK	Adenosine monophosphate-activated protein kinase
TRB3	Tribbles homologue 3
mTORC1	Mammalian target of rapamycin complex 1
DDIT4	DNA damage-inducible transcript 4
ULK1	Uncoordinated-51-like kinase 1
AMBRA1	Activating molecule in Beclin-1-regulated autophagy protein 1
PI3P	Phosphatidyl-inositol-3-phosphate
PI	Phosphatidylinositol
WIPIs	WD repeat domain phosphoinositide-interacting proteins
PE	Phosphatidylethanolamine
LAMP3	Lysosomal-associated membrane protein 3
VEGF	Vascular endothelial growth factor
TRAIL	TNF-related apoptosis-inducing ligand
Ero1	ER oxidoreductin 1
CaMK	Calcium-/calmodulin-dependent protein kinase
GADD34	Growth arrest and DNA damage-inducible protein 34
PP1	Phosphatase 1
p38MAPK	p38 mitogen-activated protein kinase
CSC	Cancer stem cells
DSF	Disulfiram
CQ	Chloroquine
HCQ	Hydroxychloroquine
EGCG	Epigallocatechin gallate
Atg5	Autophagy related protein 5
FIP200	Focal adhesion kinase family interacting protein of 200 kDa
C/EBPβ	CCAAT/enhancer-binding protein beta
EB1	End-binding protein 1
ESCRT-III	Endosomal sorting complex required for transport-III
UPR	Unfolded protein response
VEGFA	Vascular endothelial growth factor A
ROS	Reactive oxygen species
MHC	Major histocompatibility complex
LGR5	Leucine-rich repeat-containing G protein-coupled receptor 5
OLFM4	Olfactomedin 4
BCL-XL	B cell lymphoma-extra large
MCL-1	Myeloid cell leukemia-1
BAK	BCL-2 antagonist killer
BAX	BCL-2-associated X protein
DR4	Death receptor 4
DR5	Death receptor 5
BID	BH3-interacting domain death agonist
